# A Systematic Review of Cases of Acute Respiratory Distress Syndrome in the Coronavirus Disease 2019 Pandemic

**DOI:** 10.7759/cureus.8188

**Published:** 2020-05-18

**Authors:** Mizba Baksh, Virendrasinh Ravat, Annam Zaidi, Rikinkumar S Patel

**Affiliations:** 1 Internal Medicine, Dr. Nandamuri Taraka Rama Rao University of Health Sciences, Vijayawada, IND; 2 Internal Medicine, Krishna Institute of Medical Sciences, Karad, IND; 3 Medicine, Dow University of Health Sciences, Karachi, PAK; 4 Psychiatry, Griffin Memorial Hospital, Norman, USA

**Keywords:** novel corona virus, corona pandemic, severe acute respiratory syndrome coronavirus 2, mortality, hospital epidemiology, sars-cov-2 (severe acute respiratory syndrome coronavirus-2)

## Abstract

The outbreak of coronavirus disease 2019 (COVID-19) was declared a global pandemic after it spread to 213 countries and has the highest total number of cases worldwide. About 80% of COVID-19 infections are mild or asymptomatic and never require hospitalization but about 5% of patients become critically ill and develop acute respiratory distress syndrome (ARDS). The widely used management for ARDS in COVID-19 has been in line with the standard approach, but the need to adjust the treatment protocols has been questioned based on the reports of higher mortality risk among those requiring mechanical ventilation. ﻿Treatment options for this widespread disease are limited and there are no definitive therapies or vaccines until now. Although some antimalarial and antiviral drugs may prove effective against severe acute respiratory syndrome coronavirus 2 (SARS-CoV-2), their safety and efficacy are still under clinical trials. We conducted a systematic review of case reports on ARDS in SARS-CoV-2 infection to summarize the clinical presentation, laboratory and chest imaging findings, management protocols, and outcome of ARDS in COVID-19-positive patients. We need ﻿more data and established studies for the effective management of the novel SARS-CoV-2 and to reduce mortality in high-risk patients.

## Introduction and background

An outbreak of a cluster of cases of pneumonia with an unknown cause was first reported in late December 2019 in Wuhan in the Hubei Province of China. This respiratory illness during the coronavirus disease 2019 (COVID-19) is caused by a novel severe acute respiratory syndrome coronavirus 2 (SARS‐CoV‐2) [1]. COVID-19 was declared a global pandemic on January 30, 2020, after it spread to 213 countries, areas, or territories including the US, where the first case was reported on January 12, 2020 [1,2]. Community transmission of COVID-19 in the US was first reported in February that spread widely later on through close person-to-person contact via respiratory droplets, and through the infected surface to a person’s eyes, nose, or mouth [3]. As a result, active surveillance, contact tracing, quarantine, and strict social distancing were implemented worldwide to contain the transmission of the virus [1]. The overall cumulative COVID-19 incidence in the US was 119.6 cases per 100,000 population on April 7 [3].

As of April 14, 2020, there is around 600,000 total number of both confirmed and probable COVID-19 cases in the US, the highest in the world, and the total number of deaths is approximately 24,000, the majority caused by pneumonia [2]. The nationwide percentage of respiratory specimen testing positive for SARS-CoV-2, using reverse transcription-polymerase chain reaction (RT-PCR), is increasing [4]. About 80% of COVID-19 infections are mild or asymptomatic. Advanced age and underlying comorbidities are strong risk factors for severe illness, complications, and death [5]. Severe disease presentation can include dyspnea, hypoxia, with more than 50% lung involvement on imaging, whereas critically ill patients progress into respiratory failure, shock, or multiorgan system dysfunction [5]. According to a study that involved a large cohort of above 44,000 patients from China, acute respiratory distress syndrome (ARDS) develops in a median time of 8 to 12 days from illness onset [5]. Additionally, the study suggested that ARDS developed in 67%-85% of intensive care unit (ICU) patients with a mortality range as high as 72% [5].

In this systematic review based on findings of previously published case reports on ARDS in SARS-CoV-2 infection, we have comprehensively summarized the clinical presentation, laboratory and chest imaging findings, management protocols, and outcome of ARDS in COVID-19-positive patients. These case reports can provide relevant and valuable information on the minority of people with a severe presentation during the COVID-19 pandemic.

## Review

Study search strategy and selection

**Figure 1:**
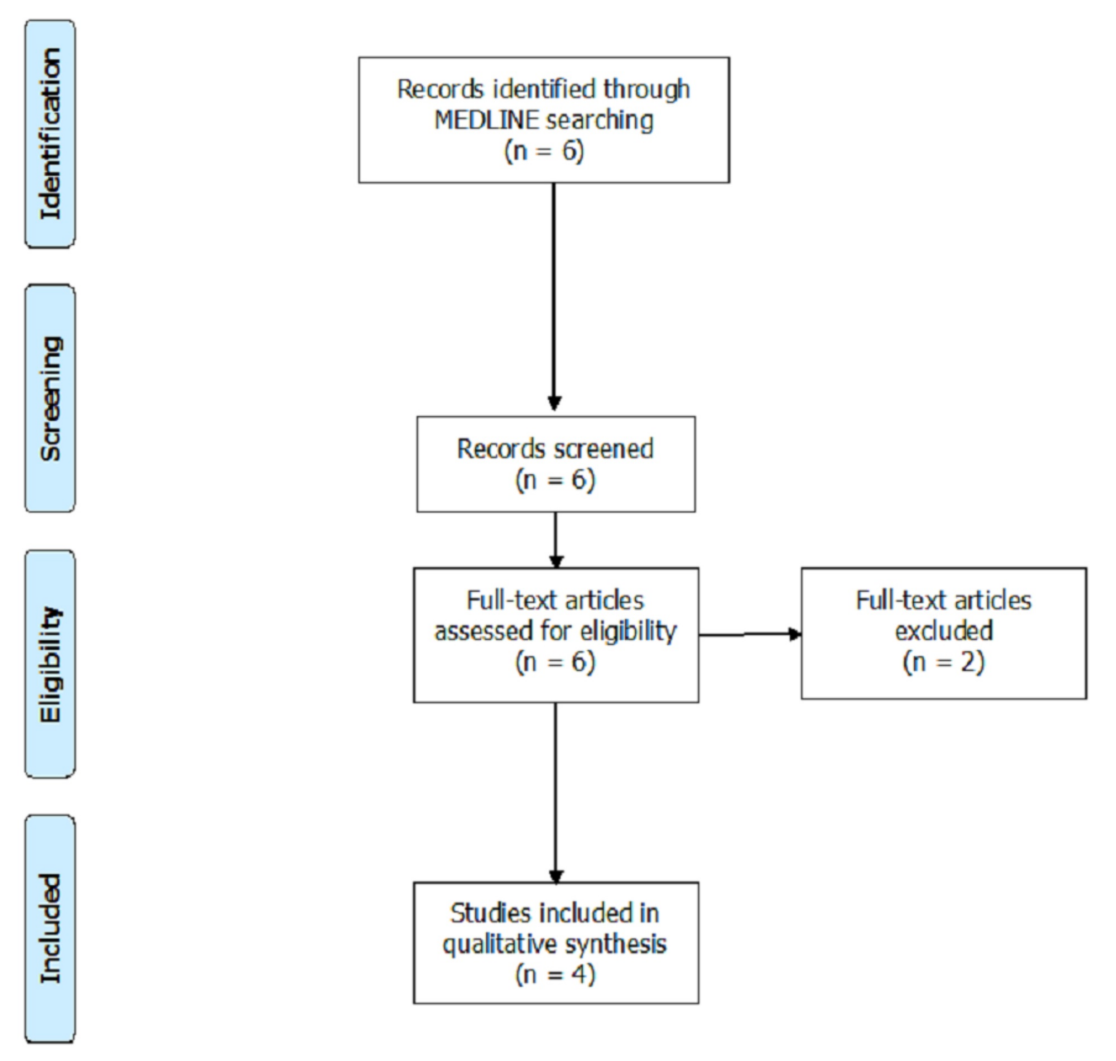
Results of the systematic review n: number of studies

The Medline database from the National Library of Medicine (NLM) was used to identify case reports published in English from December 1, 2019, to April 12, 2020. The search strings in title/abstract were COVID-19 or coronavirus and acute respiratory distress syndrome or ARDS that yielded six case reports. All searches and screening were done independently by two authors using the Preferred Reporting Items for Systematic Reviews and Meta-Analyses (PRISMA) recommendations. The initial Medline searches generated six case reports. The titles and abstracts were screened, based on the purpose of our review, and resulted in the exclusion of two case reports. A total of four case reports met the criteria for our systematic review and were included as shown in Figure 1 [6-9].

Pathophysiology

SARS‐CoV‐2 is a positive single-strand enveloped ribonucleic acid (RNA) virus that contains viral membrane E type glycoprotein that binds and enters sensitive cellular receptors by endophagocytosis in organ systems including epithelial cells in the respiratory tract [10]. The novel beta coronavirus strain that causes COVID-19 is in the same subgenus as the SARS virus of the 2003 outbreak [11]. There is only sporadic information on the pathophysiology of the disease at an early and evolving stage of the pandemic outbreak. In previous animal models and human studies on SARS pathology, it is mentioned that the SARS-CoV protein binding to angiotensin-converting enzyme 2 (ACE2) could lead to acute lung injury through ACE2 downregulation and angiotensin (AT) 1a receptor stimulation [12,13]. Animal studies found that elastase, a major protease induced in lung inflammation, might also be involved in SARS pathogenesis [12,13]. Clinical pathology of autopsy cases of SARS helped in the significant understanding of the nature of the disease. The overall pathological changes in the lungs were of diffuse alveolar damage-causing ARDS [10]. Microscopic examination of pulmonary lesions revealed extensive bilateral consolidation, hemorrhagic infarction, desquamative pulmonary alveolitis and bronchitis, hyaline membrane formation, and viral inclusion bodies in alveolar epithelial cells [10]. Imaging findings range from no abnormalities to bilateral lung consolidation on chest radiographs or peripheral ground-glass opacities on CT scan [5].

Clinical presentation

The included four case reports were published between February and April 2020; two of them were from China, one was from Singapore, and one was from the US [6-9]. A total of six patients with COVID-19 were studied for the development of critical illness and/or ARDS. Patients were adults with an age range of 44 to 75 years.

The most common initial presentation of COVID infection was a history of two to seven days of cough with or without fever, chills, dyspnea, and fatigue [6-9]. One of the patients from Iran was detected incidentally on a chest CT scan when he presented to the emergency room for follow-up of a two-week-old rib fracture from a fall with pain unresponsive to over-the-counter painkillers [9].

Disease course and outcomes

The course and development of critical illness or ARDS were similar in most cases with the patient’s condition deteriorating within 48-72 hours of initial presentation. Most of them developed dyspnea and severe hypoxemia with declining oxygen saturation (SaO_2_) during the second week of illness requiring oxygen supplementation or assisted ventilation. The patient who was diagnosed accidentally at early stages of infection was immediately treated with﻿ oseltamivir 75 mg twice daily (BID) and hydroxychloroquine 400 mg stat, based on the ﻿Iranian interim guideline for "clinical management of COVID-19", though the patient developed fever and dyspnea three days later [9]. Management was switched to a focused antiviral treatment regimen with ﻿oseltamivir 75 mg and lopinavir/ritonavir 400/100 mg BID and the patient gradually improved attaining normal oxygen saturation without the need of intubation or supplemental oxygen [9].

All six patients were tested positive for SARS-CoV-2 using the reverse RT-PCR assay of a respiratory specimen. In two of the six cases, a detailed laboratory investigation revealed lymphopenia and elevated C-reactive protein [7,8]. In one of the cases, flow cytometric analysis showed decreased peripheral cluster of differentiation (CD) 4 cells and CD8 cells [7]. Liver and renal function tests showed an elevated aspartate transaminase/alanine transaminase ratio and lactate dehydrogenase levels, and lung biopsy showed bilateral ARDS [7,8]. Three cases of ﻿critically ill, mechanically ventilated patients with ARDS required continuous monitoring of D-dimer and fibrinogen levels since it involved treatment with a fibrinolytic agent: tissue plasminogen activator (tPA) [6]. Chest x-ray and chest CT scan on admission showed predominant bi-basilar ground-glass opacities in all six patients [6-9].

Management

Treatment modalities and clinical management options for COVID-19-induced ARDS were variable among these patients but mainly supportive and similar to standard ARDS management. Infection control measures that included patient placement in isolation wards and standard contact and airborne precautions were pre-requisite. Oxygen supplementation was a standard protocol for most patients who developed dyspnea and hypoxemia. Antiviral therapy was tried in three of the six cases mentioned either as lopinavir/ritonavir 500 mg BID or in combination with oseltamivir as 400/100 mg BID [7-9]. One patient died of hypoxemia and sudden cardiac arrest (patient was on the do-not-resuscitate code status), but the other two showed marked improvement after receiving treatment [7-9]. The other medication commonly used (in four of six cases) was hydroxychloroquine stat 400 mg, in combination with either azithromycin or oseltamivir (75 mg) [6,9]. Empiric broad-spectrum antibiotics such as moxifloxacin were used in two mechanically ventilated patients to prevent secondary infection; however, one patient developed ventilator-associated pneumonia that necessitated the use of culture-guided antibiotics [7,8]. Corticosteroids, such as intravenous methylprednisone, were administered in one patient to decrease lung inflammation [7].

The study on tPA treatment for COVID-19-associated ARDS, which involved measuring ﻿the partial pressure of oxygen (PaO_2_)/fraction of inspired oxygen (FiO_2_) ratio for oxygenation status, reported one out of three cases had 100% improvement post-tPA but the effect was transient [6]. This case series also mentions the use of anticoagulants like heparin with tPA infusion to decrease the risk of bleeding [6]. There are few in vivo studies on the use of plasminogen activators for the prevention of acute lung injury in animal studies, and so more trials are required to determine the optimal dosing and therapeutic effects of tPA [14,15]. Vasopressors such as norepinephrine, phenylephrine, and vasopressin have been proved effective for hemodynamic support, ﻿sedation, and chemical paralysis [15-17]. Few studies have summarized the use of non-ventilatory interventions as rescue therapy in non-compliant patients with refractory hypoxemia [16,17]. A descriptive summary of all case reports that met our inclusion criteria is shown in Table 1.

**Table 1:** Summary of included case reports of ARDS in COVID-19 patients AST/ALT: aspartate transaminase/alanine transaminase; LDH: lactate dehydrogenase; SOB: shortness of breath; b/l: bilateral; CXR: chest x-ray; SARS-CoV-2: severe acute respiratory syndrome coronavirus 2; COVID-19: coronavirus disease 2019; CD: cluster of differentiation; BID: twice daily; HFNC: high-flow nasal cannula; ARDS: acute respiratory distress syndrome; Rx: treatment; PaO_2_/FiO_2_: partial pressure of oxygen/fraction of inspired oxygen; OTC: over the counter; tPA: tissue plasminogen activator; ED: emergency department; CRRT: ﻿continuous renal replacement therapy; PTT: partial thromboplastin time; SpO_2_: peripheral oxygen saturation; SaO_2_: oxygen saturation of arterial blood; PEEP: positive end expiratory pressure; RT-PCR: reverse transcription-polymerase chain reaction; RR: respiratory rate; CRP: C-reactive protein; IL-6: interleukin-6; NRB: non-rebreather mask; DNR: do not resuscitate; HEPA: high-efficiency particulate air

Study	Demographics	Initial presentation	Development of ARDS	Imaging studies	Lab findings	Treatment	Prognosis and outcome/mortality
Xu et al. [7]	50-year-old Asian male	COVID-19 mild symptoms with chills and dry cough	Patient developed severe symptoms with fever, chills, fatigue, and SOB leading to hospitalization	Initial CXR showed multiple patchy shadows in b/l lungs. CXR on day 12 showed progressive infiltrate and diffuse gridding shadow in b/l lungs	PCR assay confirmed COVID-19. Lung biopsy showed bilateral diffuse alveolar damage with exudates, b/l lung showed evident ARDS. Biopsy of heart tissue showed no substantial damage. Liver biopsy showed injury caused by SARS-CoV-2 infection or drug toxicity. Flow cytometry showed an increase of Th17 and high cytotoxicity of CD8 T cells. Lymphopenia with reduced peripheral CD4 and CD8 T cells, elevated AST/ALT ratio and elevated LDH, hypoalbuminemia, elevated IL-6 and CRP	Supplemental oxygen, interferon alfa-2b 5 million units BID via atomization inhalation, lopinavir plus ritonavir 500 mg BID as antiviral therapy, moxifloxacin 0.4 g daily IV to prevent secondary infection, methylprednisolone 80 mg twice daily IV for SOB	Day 12: CXR showed progressive infiltrate and the patient refused ventilator support, so received HFNC oxygen (60% concentration, flow rate 40 L/min). Day 14: hypoxemia and SOB worsened despite receiving HFNC oxygen therapy (100% concentration) with oxygen saturation decreased to 60%. Patient had a sudden cardiac arrest and died even after receiving invasive ventilation, chest compression, and adrenaline
Goh et al. [8]	64-year-old Asian (Singaporean) male	Presented with a fall preceded by dizziness and reported a one-week history of fever and a one-day history of dyspnea	Within 48 hours of presentation, the patient deteriorated rapidly with severe hypoxemic respiratory failure. His vitals were stable except RR of 18-20 breaths/min	CXR on admission showed subtle ground-glass opacities in the right lung and atelectasis in the left. No consolidation or pleural effusion was seen. CT scan thorax on day 8 revealed diffuse ground-glass opacities and consolidation in both lungs consistent with ARDS	﻿RT-PCR was positive for SARS-CoV-2. Lab investigations showed lymphopenia, elevated CRP, and normal to slightly elevated procalcitonin. Renal and liver function tests and serum lactate on admission were normal. Stool PCR was positive for SARS-CoV-2 and Clostridium difficile toxin assays were negative when tested for diarrhea	Isolated in an airborne infection isolation room, lopinavir/ritonavir started on day 2 of hospitalization, empirical broad-spectrum antibiotics were started on day 2 of mechanical ventilation, antibiotics on day 10 for ventilator-associated pneumonia	Day 3: patient was intubated with mechanical ventilation and HEPA filter. He developed moderate to severe ARDS (PaO_2_/FiO_2_ 114) and significant ventilator dys-synchrony, so neuromuscular blockade was initiated to maintain lung-protective ventilation. The patient did not require prone ventilation and fully recovered 4 days later. Day 10: his ventilatory requirements increased and new consolidation developed on chest radiograph. He was given antibiotics for ventilator-associated pneumonia and successfully extubated on day 14. First negative PCR only achieved on day 15 (3 weeks from symptom onset) followed by resolution of lymphopenia
Asadollahi-Amin et al. [9]	44-year-old Iranian (Persian) male	Presented to ED with a history of rib fracture from a fall about two weeks ago and persistent local tenderness and pain even after taking OTC painkillers	Lung involvement was found incidentally on chest CT done for rib fracture. After 3 days of Rx with oseltamivir and hydroxychloroquine on testing positive for SARS-CoV-2, the patient developed fever and dyspnea	Chest CT revealed left 8th and 9th ribs fracture and ﻿an ill-defined patchy ground-glass opacity in the right lung	RT-PCR assay confirmed the diagnosis of COVID-19 infection	Oseltamivir 75 mg BID and hydroxychloroquine ﻿400 mg stat were given based on Iranian interim guidelines for COVID-19, oseltamivir 75 mg and lopinavir/ritonavir ﻿400/100 mg BID. Rx regimen was changed after 3 days of previous Rx as fever and dyspnea developed	﻿After approx. 24 hours of changed focused therapy, patient's clinical condition started to improve, his ﻿O_2_ saturation increased to 97%, and on day 5, patient became asymptomatic
Wang et al. [6]	75-year-old male	One week of cough, fatigue, and fevers	Respiratory vital signs on presentation were abnormal: RR 22 and SpO_2_ 91% requiring 100% FiO_2_ on NRB by day 3 of hospitalization	CT chest showed b/l ground-glass opacities mainly in peripheral and basal areas	COVID-19 testing was positive. D-dimer levels were consistently elevated for 4 days following intubation but decreased post-tPA. Fibrinogen levels were also slightly elevated	﻿Hydroxychloroquine and azithromycin for 5 days. ﻿CRRT was initiated for anuria tPA 25 mg IV over 2 hours, followed by 25 mg tPA infusion over the next 22 hours, ﻿heparin infusion was started at 10 units/kg/hour with a PTT goal of 60-80, vasopressors such as norepinephrine, phenylephrine, and vasopressin were used	Day 6: ﻿severe hypoxemia persisted with PaO_2_/FiO_2_ ratio was 73 leading to intubation and SaO_2_ improved from 85% to 91% in prone position. Day 8: ﻿patient became anuric ﻿with persistently elevated D-dimer, so tPA was given which he tolerated well. His PaO_2_/FiO_2_ ratio worsened to 136, 1 hour into heparin infusion but 48 hours post-tPA ﻿his PaO_2_/FiO_2_ improved to 188-250. Day 11: patient developed multi-organ failure with refractory hypotension secondary to atrial fibrillation and superimposed bacterial infection. He died as he was made DNR
	59-year-old female	Two days of rhinorrhea, cough, myalgias, and headaches	Vital signs from her initial presentation were not available. Patient developed severe hypoxemia requiring 100% NRB after two days of O_2_ supplementation	CT chest demonstrated bibasilar ground-glass opacities	COVID-19 testing was positive. D-dimer increased dramatically from day 6 to day 9 and achieved the highest levels of 12 hours of post-tPA infusion. Fibrinogen levels were also elevated	Hydroxychloroquine and azithromycin started on the diagnosis of COVID-19. One vasopressor was used for hemodynamic support, ﻿sedation, and chemical paralysis, ﻿IV tPA 25 mg intravenous bolus over 2 hours, followed by 25 mg tPA infusion over the subsequent 22 hours, heparin infusion post-tPA	Day 4: ﻿she was transferred to hospital for intubation for hypoxemic respiratory failure. PaO_2_/FiO_2_ ratio improved to 130s in prone position. After 4 days of intubation and 12 hours post-tPA, PaO_2_/FiO_2_ ratio improved to 150s (prone), but 38 hours post-tPA, the ratio was 135 in supine position (was 90 pre-tPA)
	49-year-old male	﻿Six days of cough, progressive dyspnea, fever, and myalgias	Vital signs were ﻿RR of 24 and SpO_2_ was 40% on room air in ED	CT chest showed ﻿bibasilar ground-glass opacities	﻿D-dimer reduced on day 2 and again increased post-tPA. Fibrinogen decreased 35 hours post-tPA	﻿Hydroxychloroquine and azithromycin were started as well as heparin drip for suspicion of venous thromboembolism, IV tPA and heparin drip resumed after tPA completion	Patient's FiO_2_ improved on 100% NRB but was intubated due to increasing tachypnoea and PEEP had to be reduced to <20 as the patient developed pneumopericardium. His PaO_2_/FiO_2_ ratio kept improving in prone position for 3 hours post-tPA, but declined 33 hours post-tPA and had to be placed back in prone position for recovery

Current evidence on treatment

About 80% of patients with COVID-19 have mild disease and never require hospitalization, and about 5% of patients become critically ill, with the risk of ARDS being highest in ICU patients [5,18]. There could be a high risk of mortality (about two-thirds) in ventilated patients according to new data from the United Kingdom’s Intensive Care National Audit and Research Center (ICNARC), but this was unclear [19]. Other less frequent complications include acute cardiac injury, acute kidney injury, and septic shock, followed by multi-organ failure [20]. Of the six patients in our review, two died from complications within one to two weeks of clinical presentation [6,7]. The reported causes of death included cardiac arrest even after receiving invasive ventilation and chest compression and the other patient in Wang et al.'s study died due to multi-organ failure with secondary bacterial infection [6,7]. The other four patients showed a good prognosis with no inpatient death.

Antiretroviral protease inhibitors, such as lopinavir/ritonavir, and antimalarials like hydroxychloroquine, for which US Food and Drug Administration (FDA) has issued an emergency use authorization (EUA), were used in all studies but randomized clinical trials (RCTs) to assess their efficacy and safety are still ongoing [21]. Sanders et al. suggested that remdesivir can be a promising therapy for COVID-19 as it has already shown broad antiviral activity in both in vitro and in vivo studies against related viruses: Middle East respiratory syndrome (MERS)-CoV and SARS-CoV [22]. Oseltamivir has no role in COVID-19 treatment and corticosteroids that have been widely used in many patients in China may potentially prolong the course of illness by causing delayed viral clearance [22]. Antimalarial drugs like chloroquine or hydroxychloroquine monotherapy or combination therapy with azithromycin may prove effective, especially in severe disease, but ﻿these benefits need to be determined with RCTs that are already underway [22,23]. So ﻿treatment options are limited and there are no definitive therapies or vaccines until now and additional studies are needed to evaluate their effectiveness [22].

According to previous reports from China and new ICNARC findings from England, mortality was higher among those requiring mechanical ventilation than those who did not and appears higher than that for patients treated in ICU for other types of viral pneumonia [19,24]. The widely used management for ARDS in COVID-19 has been in lines with the standard approach, but treatment protocols need to be adjusted according to the characteristics of disease pathophysiology, making more gradual positive end-expiratory pressure changes for the atypical type of ARDS seen with COVID-19 [19].

## Conclusions

Our systematic review of published cases of ARDS in COVID-19-positive patients will help healthcare professionals to clearly understand and implement updated treatment strategies and confront the COVID-19 pandemic and its medical consequences. Nonetheless, we need ﻿more RCTs and treatment guidelines for developing effective management of the novel SARS-CoV-2 and thus improve survival and reduce mortality in high-risk and critical patients.
